# Supplementary material for: Novel Peritoneal Sclerosis Rat Model Developed by Administration of Bleomycin and Lansoprazole
Source: Int J Mol Sci. 2023 Nov 9;24(22):16108. doi: 10.3390/ijms242216108 (PMC10671295; doi:10.3390/ijms242216108)
Supplement: Supplementary file 1 [file ijms-24-16108-s001.zip › ijms-2670738-supplementary.pdf]

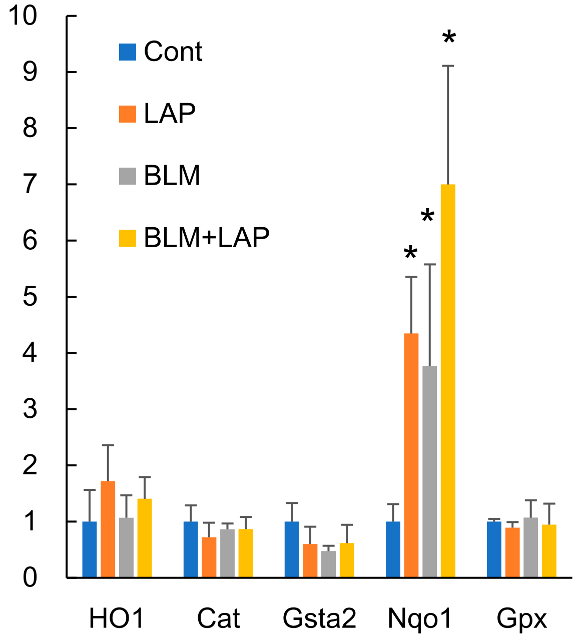

Supplemental Figure S1. The effect of lansoprazole and/or bleomycin for four weeks on expression changes of antioxidative stress proteins.

The expression changes of anti-oxidative stress protein genes were measured. Asterisks indicate significant difference compared with the control ($P < 0.05$). Each five rats were allocated in control, lansoprazole, or bleomycin, and six rats were allocated in BLM+LAP groups. *P* values were calculated by Dunnet's test. LAP: lansoprazole, BLM: bleomycin.

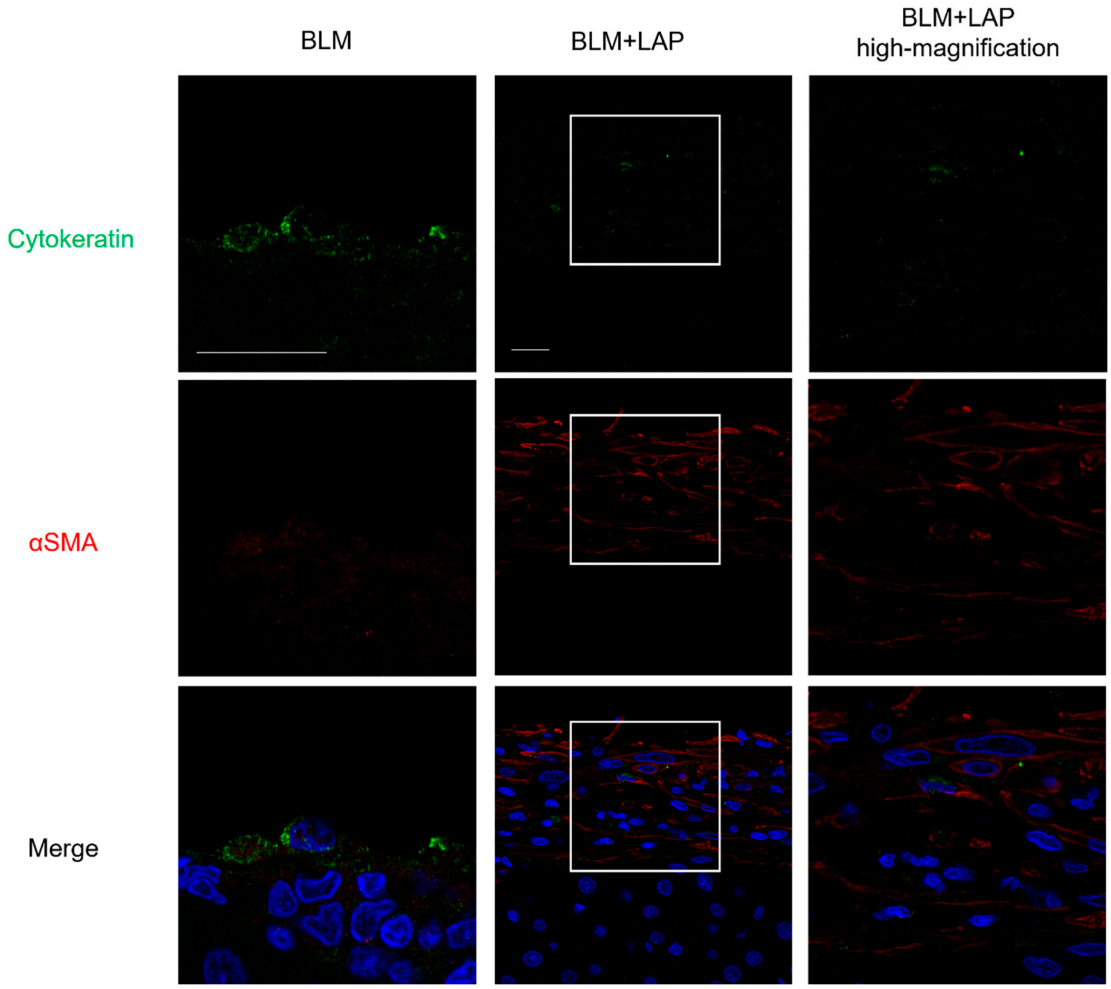

Supplemental Figure S2. Administration of BLM+LAP was associated with mesothelial-mesenchymal transition.

In one out of five rats from BLM group, reactive mesothelial cells were detected. Immunoreactivity against cytokeratin was detected and it against αSMA was slightly detected in the reactive mesothelial cells from BLM group. The immunoreactivity against cytokeratin was weak in a case in which the peritoneal tissue was thickened from BLM+LAP group. Scale bar indicates 60 μm.

Supplemental Table S1. Result of two-way ANOVA in the expression of chemokines

	Lansoprazole		Bleomycin		Interaction	
	F	P	F	P	F	P
2w outer part						
Ip10	0.48	0.50	5.05	0.04	0.95	0.34
Mip1a	8.04	0.01	10.86	0.00	15.44	0.00
Mcp1	0.00	0.97	0.01	0.94	2.26	0.15
Mcp3	0.00	0.99	0.00	0.99	2.26	0.15
Rantes	0.51	0.49	1.58	0.23	0.01	0.93
4w outer part						
Ip10	2.80	0.11	0.17	0.68	0.79	0.39
Mip1a	3.53	0.08	1.46	0.24	1.44	0.25
Mcp1	0.00	0.98	0.63	0.44	9.47	0.01
Mcp3	0.00	0.98	1.41	0.25	2.52	0.13
Rantes	0.94	0.35	0.00	0.97	1.08	0.32
2w inner part						
Ip10	3.08	0.10	4.79	0.04	3.10	0.10
Mip1a	8.62	0.01	5.72	0.03	13.18	0.00
Mcp1	5.24	0.04	7.69	0.01	8.87	0.01
Mcp3	0.44	0.52	0.30	0.59	1.49	0.24
Rantes	0.04	0.84	5.05	0.04	2.28	0.15
4w inner part						
Ip10	2.15	0.16	0.00	0.99	1.26	0.28
Mip1a	1.32	0.27	1.31	0.27	0.58	0.46
Mcp1	0.06	0.82	0.00	0.95	0.09	0.77
Mcp3	0.05	0.82	0.17	0.69	0.00	0.99
Rantes	0.26	0.62	0.06	0.80	0.78	0.39
